# Comparative Study of Cytotoxic and Membranotropic Properties of Betulinic Acid-F16 Conjugate on Breast Adenocarcinoma Cells (MCF-7) and Primary Human Fibroblasts

**DOI:** 10.3390/biomedicines10112903

**Published:** 2022-11-11

**Authors:** Konstantin N. Belosludtsev, Anna I. Ilzorkina, Natalia V. Belosludtseva, Vyacheslav A. Sharapov, Nikita V. Penkov, Dmitriy A. Serov, Maxim N. Karagyaur, Darya A. Nedopekina, Eldar V. Davletshin, Marina E. Solovieva, Anna Yu Spivak, Ulyana Sh. Kuzmina, Yulia V. Vakhitova, Vladimir S. Akatov, Mikhail V. Dubinin

**Affiliations:** 1 Department of Biochemistry, Cell Biology and Microbiology, Mari State University, pl. Lenina 1, 424001 Yoshkar-Ola, Russia; 2 Institute of Theoretical and Experimental Biophysics, Russian Academy of Sciences, Institutskaya 3, 142290 Pushchino, Russia; 3 Federal Research Center, Pushchino Scientific Center for Biological Research of the Russian Academy of Sciences, Institute of Cell Biophysics of the Russian Academy of Sciences, Institutskaya 3, 142290 Pushchino, Russia; 4 Prokhorov General Physics Institute of the Russian Academy of Sciences, Vavilov St. 38, 119991 Moscow, Russia; 5 Institute for Regenerative Medicine, Medical Research and Education Center, Lomonosov Moscow State University, 27/10, Lomonosovsky Ave., 119192 Moscow, Russia; 6 Faculty of Medicine, Lomonosov Moscow State University, 27/1, Lomonosovsky Ave., 119192 Moscow, Russia; 7 Institute of Petrochemistry and Catalysis, Ufa Federal Research Center, Russian Academy of Sciences, Prospekt Oktyabrya 141, 450075 Ufa, Russia; 8 Institute of Biochemistry and Genetics, Ufa Federal Research Center, Russian Academy of Sciences, Prospekt Oktyabrya 71, 450054 Ufa, Russia

**Keywords:** F16, delocalized lipophilic cation (DLC), BA, oxidative stress, cytotoxicity, breast cancer

## Abstract

The present study evaluates the cytotoxicity of a previously synthesized conjugate of betulinic acid (BA) with the penetrating cation F16 on breast adenocarcinoma (MCF-7) and human fibroblast (HF) cell lines, and also shows the mechanism underlying its membranotropic action. It was confirmed that the conjugate exhibits higher cytotoxicity compared to native BA at low doses also blocking the proliferation of both cell lines and causing cell cycle arrest in the G_0_/G_1_ phase. We show that the conjugate indeed has a high potential for accumulation in mitochondria, being visualized in these organelles, which is most pronounced in cancer cells. The effect of the conjugate was observed to be accompanied by ROS hyperproduction in both cancerous and healthy cells, despite the lower base level of ROS in the latter. Along with this, using artificial liposomes, we determined that the conjugate is able to influence the phase state of lipid membranes, make them more fluid, and induce nonspecific permeabilization contributing to the overall cytotoxicity of the tested agent. We conclude that the studied BA–F16 conjugate does not have significant selective cytotoxicity, at least against the studied breast cancer cell line MCF-7.

## 1. Introduction

Natural substances have played an increasing role in the discovery and development of new drugs. Moreover, most of the antitumor and anti-infective drugs on the market to date are of natural origin [1]. Therefore, the identification of natural plant compounds for disease control has become a popular avenue of research in biology and medicine.

Triterpenoids are one of several groups of phytochemicals with promising biological properties. Triterpenoids belong to terpenoids (also known as isoprenoids), the largest group of natural products [2]. These compounds are composed of six isoprene units and can be isolated from many different plant sources.

Betulinic acid (BA), or 3*β*-3-hydroxy-lup-20(29)-en-28-oic acid (BA), is a naturally occurring pentacyclic triterpenoid of the lupane series [3]. It is found both in the bark of birches (Betula sp., Betulaceae) and in other plant genera: Ziziphus (Rhamnaceae), Sizigium (Myrtle), Diospyros (Ebony), and Paeonia (Paeoniaceae). The compound is present in the plant as a free aglycone or as glycosylated derivatives [4] that are easily isolated using various solvents, most often dichloromethane [5].

In recent decades, numerous studies have been conducted on the biological activity and medicinal properties of BA. At present, antitumor, antiatherogenic, anti-inflammatory, antioxidant, anti-HIV, hepatoprotective, and antidiabetic properties are attributed to BA [6]. At the same time, all native natural triterpenoids of the lupane series have a significant disadvantage, which hinders their use in medicine. This concerns low bioavailability due to the poor solubility of triterpenoids in biological fluids limiting their effective interaction with biological targets [7]. Therefore, to achieve the desired therapeutic effect, it is necessary to use high concentrations of these compounds, which, in turn, is fraught with various side effects. Therefore, the efforts of many laboratories are aimed at determining the methods for the chemical modification of triterpenoids to increase their bioavailability and activity [8].

**Figure 1:**
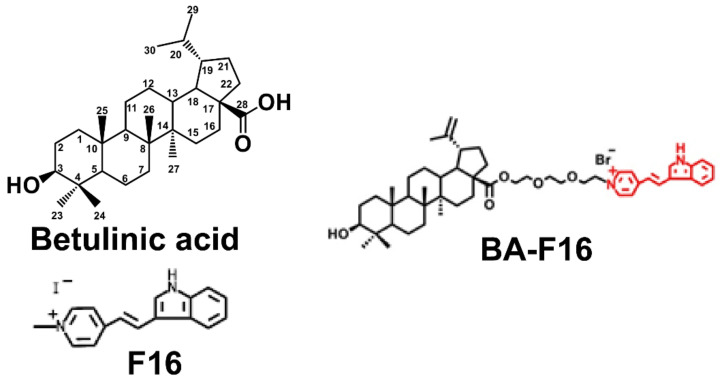
Structure of BA (BA), F16 (E-4-(1H-indol-3-ylvinyl)-N-methylpyridinium iodide]), and BA–F16 conjugate [17].

One of the popular directions in the field of increasing the bioavailability of native triterpenoids is the creation of synthetic hybrids based on triterpenes and specific groups that impart targeted properties, which is used, in particular, in the creation of antitumor agents targeting mitochondria (the main cell organelles involved in maintaining cell metabolism and cell death regulation). The main chemical agents successfully used to create hybrid molecules that can target mitochondria include delocalized lipophilic cations (DLCs), such as rhodamine 123, F16, MKT-077, and lipophilic cationic salts of dequalinium and triphenylphosphonium [9,10,11,12,13,14,15,16]. DLCs are a family of compounds that have the ability to cross plasma and mitochondrial membranes, accumulating in mitochondria due to a negative transmembrane potential. Recently, we synthesized conjugates of BA and the lipophilic cation F16 (E-4-(1H-indol-3-ylvinyl)-N-methylpyridinium iodide), which showed a significant increase in the cytotoxic effect compared to the original BA (≈100–200 times) [17]. The synthesized compounds reduced the membrane potential, suppressed the process of respiration and oxidative phosphorylation, and increased the rate of generation of reactive oxygen species (ROS) in isolated rat liver mitochondria [18]. The results obtained suggest that the cytotoxic effects underlying the mechanisms of action of BA-F16 conjugates may be associated with the mitochondria-targeted nature of these compounds. In this case, one of the most active conjugates was the compound formed by the covalent binding of BA to the F16 cation using a triethylene glycol spacer (BA-F16) (Figure 1). According to our previous studies, this compound exhibited increased cytotoxicity against some cancer cell lines, and, in contrast to other compounds, induced the uncoupling of respiration and oxidative phosphorylation in mitochondria and permeabilization of artificial membranes [17,18].

In this work, we continue our studies of the mechanisms of the cytotoxic action of the BA–F16 conjugate. We study the cytotoxic effect of BA and BA–F16 on MCF-7 cancer cells and human fibroblasts, and evaluate the effect of these compounds on the cell cycle and the production of superoxide anion. In parallel, we assess the degree of localization of BA–F16 in the mitochondria of both cell lines. In conclusion, we observe that the BA–F16-dependent non-specific increase in the permeability of liposomal membranes may be associated with a change in the degree of ordering of lipid bilayer membranes.

## 2. Materials and Methods

### 2.1. Reagents and Chemicals

BA, propidium iodide (PI), fetal bovine serum (FBS), human insulin, and doxorubicin were purchased from Sigma-Aldrich (St. Louis, MO, USA). Dulbecco’s Modified Eagle Medium (DMEM) containing 1 g/L glucose, 25 mM HEPES, sodium bicarbonate; L-glutamine; gentamicin; Hank’s solution; and 0.25% trypsin-EDTA solution were purchased from Paneco (Moscow, Russia). Phosphatidylcholine was purchased from Avanti Polar Lipids (Avanti Polar Lipids Inc., Alabaster, AL, USA). The conjugate and BA used in this study were prepared in DMSO as a 5–10 mM stock solution.

### 2.2. Synthesis of BA–F16 Conjugate

The synthesis of the derivative was performed in accordance with the previously tested method [17]. The structure of the product was confirmed by 1D (^1^H, ^13^C, APT) and 2D homo- (COSY, NOESY) and heteronuclear (HSQC, HMBC) NMR experiments. The nuclear chemical shifts for the terpene core and (E)-4-(1H-indol-3-ylvinyl)pyridine were determined by comparison with the published data [19] and for the E-4-1H-Indol-3-ylvinyl pyridinium moiety [17,20] in the original paper and Supplementary Materials. In the ^1^H NMR spectra, the presence of the (E)-4-(1H-indol-3-ylvinyl)pyridinium moiety was evidenced by 2 characteristic doublets for the pyridine ring at 8.02 and 8.58 ppm with J = 6.0 Hz, 2 vinyl-group doublets at 7.10 and 8.09 ppm with J = 16.0 Hz, and 3 characteristic multiplets for the indole moiety at 7.22–7.29, 7.48–7.88, and 8.07–8.09 ppm. The ^13^C NMR spectra presented signals for the (E)-4-(1H-indol-3-ylvinyl)pyridinium carbons in the 113.6–157.4 ppm range.

### 2.3. Cell Culture Conditions

The human breast adenocarcinoma MCF-7 cell line was obtained from the Russian collection of cell cultures (Institute of Cytology RAS, St Petersburg, Russia). Human primary dermal fibroblasts (HFs) derived from a patch of skin from healthy donors (*n* = 3) were obtained from the biobank at the Institute for Regenerative Medicine, Lomonosov Moscow State University, collection ID: MSU_FB (https://human.depo.msu.ru, accessed on 14 September 2021). The cells were cultured in Dulbecco’s Modified Eagle Medium (DMEM) Low Glucose (#11885084, Gibco, Grand Island, NY, USA) containing 10% of fetal bovine serum (#SH30071.02HI, HyClone, South Logan, UT, USA), 1% of Antibiotic Antimycotic (Pen/Strep/Fungizone) solution (#SV30079.01, HyClone, South Logan, UT, USA), and 1 mg/mL of insulin (MCF-7).

### 2.4. Cytotoxicity Study

To analyze the cytotoxic effect, MCF-7 cells were seeded in 96-well Nunc culture plates (Thermo Scientific) at a concentration of 12 × 10^4^ cells/mL (12 × 10^3^ cells in 100 µL of nutrient medium per well) and at a concentration of 35 × 10^3^ cells/mL (3.5 × 10^3^ cells in 100 µL of culture medium per well) for HF cells. The additions of conjugate and BA or BA-F16 were performed in triplicate from freshly prepared solutions of the initial conjugate (5–10 mM) in Hank’s solution at concentrations of 0.01–100 µM. The final concentration of DMSO in the freshly prepared conjugate solution did not exceed 1% and was not toxic to the cells. All treatments were performed 24 h after seeding the cells on the plates. For each concentration, the experiments were performed in triplicate. The incubation of cells with conjugates lasted 48 h. Cytotoxicity was determined using the method of determining the number of cells in a culture monolayer using crystal violet (CV) dye. It was based on the binding of CV to nucleoproteins in fixed cells, and the amount of bound dye correlated linearly to the number of viable cells. To perform this, following incubation, the medium was removed from the plates, and the cells were stained with a 0.2% alcohol solution of CV, incubated with the dye, washed with water, and dried. After 24 h, SDS was added to each well. The cytotoxicity of the compounds was determined by the ratio of optical densities minus the measured background absorbance in treated and untreated cultures 48 h after the addition of the compounds using an Infinite 200 microplate spectrofluorimeter (TECAN, Grödig, Austria). The optical density value was directly proportional to the number of viable cells. The number of viable cells was assessed using trypan blue exclusion assay following cell culture trypsinization. The concentration value that caused a 50% inhibition of cell population growth (IC_50_) was determined from dose-dependent curves.

### 2.5. Flow Cytometry Assay

To analyze the cell cycle, membrane potential, and presence of oxidative stress, the cells were seeded in T-25 flasks or P-35 Petri dishes for cultivation (Corning, New York, NY, USA) at a concentration of 12 × 10^4^ cells/mL for MCF-7 and at a concentration of 35 × 10^3^ cells/mL for HF. Conjugate and BA were added at concentrations of 1 and 5 μM, respectively, 24 h after cell seeding, and incubated for 24 or 48 h at 37 °C in a humidified atmosphere containing 5% CO_2_. Following incubation, the cells were collected by trypsinization together with the medium and washed with ice-cold PBS buffer (pH 7.4). Then, the resulting cells were resuspended in PBS buffer, 2 volumes of 70% cold ethanol were added to the tubes, and mixed with the cells by slow stirring. After fixing with ethanol at −20 °C overnight, the cells were centrifuged (1.0 × 1000 rpm, 3 min) and the supernatant was discarded. Each cell sample was then resuspended in PBS and stained with the fluorescent DNA-binding dye propidium iodide (10 μg/mL). Subsequently, 50 μg/mL of RNase A (Sigma-Aldrich, St. Louis, MO, USA) was added to each sample, and the resulting samples were incubated at 37 °C for 30 min. The distribution of the cells between G_0_/G_1_, S, and G_2_/M phases of the cell cycle were evaluated using BD Accuri C6 (BD Bioscience, San Jose, CA, USA), and the obtained data were analyzed using ModFit LT 4.1 software.

To assess the level of oxidative stress, the cells, after cultivation and incubation (as described above), were collected by trypsinization together with the medium, and washed with ice-cold PBS buffer (pH 7.4). The resulting suspension was centrifuged (1000 rpm, 5 min). The precipitated cells were then resuspended in PBS buffer. Oxidative stress cell populations were quantified using the Muse Oxidative Stress Kit (MCH100111) and Muse Cell Analyzer (Merck Millipore, Burlington, MA, USA). All analyses were performed according to the manufacturer’s protocols.

### 2.6. Assessment of Mitochondrial Localization of BA Conjugate

The study of the mitochondrial localization of BA-F16 was performed using the method of confocal microscopy. A total of 3 days before the start of the experiments, the cells were subcultured on round coverslips. The resulting cells were washed from the culture medium and placed in complete Hank’s solution. Then, 200 nM MitoTracker DeepRed FM (ThermoFisher, Waltham, MA, USA) and the test compound (at a final concentration of 200 nM) were added to the samples. The samples were incubated for 30 min at 37 °C in the dark, then the cells were washed twice with fresh Hank’s solution. Confocal images were obtained using a DMI6000 microscope (Leica, Germany) equipped with a 63× oil immersion objective. MitoTracker DeepRed FM fluorescence was measured at wavelengths of (Ex/Em) 638/650 nm; conjugate at 450/550 nm. The maximums were indicated for emission (window 20 nm). Colocalization was assessed using ImajeJ 1.52p (Fiji) software (NIH, Bethesda, MD, USA).

### 2.7. Liposome Preparation

Liposomes (large unilamellar vesicles, LUVs) were obtained by the conventional extrusion method [21]. Dry dipalmitoylphosphatidylcholine (DPPC, 7.5 mg) was hydrated in 0.75 mL of buffer with the addition of a laurdan fluorescent probe (7 μL) for several hours with occasional mixing in a vortex mixer. The buffer contained 10 mM Tris/HCl (pH 7.5), 40 mM KCl, and 50 μM EGTA. After 5 freeze/thaw cycles at −20/+ 55 °C, the multilamellar liposome suspension was subjected to 11 extrusion steps through a polycarbonate membrane (0.1 µm pore diameter) using an Avanti microextruder (Avanti Polar Lipids, Birmingham, AL, USA).

### 2.8. Measurement of Permeabilization of SRB-Loaded Liposomes

SRB-loaded LUVs were prepared from egg-phosphatidylcholine by a procedure similar to that described above, except that (1) the buffer for lipid hydration contained 50 mM of SRB instead of 40 mM KCl, and (2) after extrusion, the liposomes were applied on a Sephadex G-50 column to remove the external SRB. The buffer for gel filtration contained 10 mM Tris/HCl (pH 8.5), 50 μM EGTA and 40 mM KCl. The release of SRB was evaluated by the increase in its fluorescence, as previously described [21]. The medium contained 10 mM Tris/HCl (pH 7.5), 50 μM EGTA, and 40 mM KCl. The lipid concentration was 25–30 μM. Fluorescence was measured using an Ocean Optics FLAME-T-UV-VIS fiber-optic system (Ocean Optics Inc., Dunedin, FL, USA) (excitation at 565 nm, emission at 586 nm). The total release of the dye was evaluated by the addition of 0.1% Triton X-100. The concentration of SRB in the external buffer was calculated using a calibration curve.

### 2.9. Measurement of the Generalized Laurdan Polarization (GP Parameter)

Laurdan is an environment-sensitive fluorescent probe, which is conventionally used for monitoring phase state of lipid membranes. We measured laurdan fluorescence (λ_ex_ = 360 nm) using a Cary Eclipse spectrofluorimeter. Emission wavelengths, corresponding to the blue and red peaks of laurdan in DPPC liposomes, were 440 and 490 nm. The generalized polarization (GP) was defined as GP = (I_440_-I_490_)/ (I_440_+I_490_) where I_440_ and I_490_ are the emission intensities at 440 and 490 nm, respectively [22]. GP can theoretically assume values from +1 (being most ordered) and −1 (being least ordered). In the experiments with laurdan-containing DPPC liposomes (laurdan/lipid molar ratio 1:200), the suspension of vesicles was added to a buffer containing 40 mM KCl, 50 μM EGTA and 10 mM Tris/HCl (pH 7.5) (the final concentration of lipid 50-60 μM), and laurdan fluorescence was measured before and after various experimental additions.

### 2.10. Statistical Analysis

The data were analyzed using GraphPad Prism 8 and Excel software, and were presented as means ± SEM. The statistical significance of the differences between the means was evaluated using the one-way analysis of variance (ANOVA) followed by Tukey’s multiple comparison post hoc test, and *p* < 0.05 was selected as an indicator of statistical significance.

## 3. Results

### 3.1. Conjugation of BA with the Penetrating Cation F16 Leads to an Increase in the Cytotoxic Effect of BA to MCF-7 and HF

**Figure 2:**
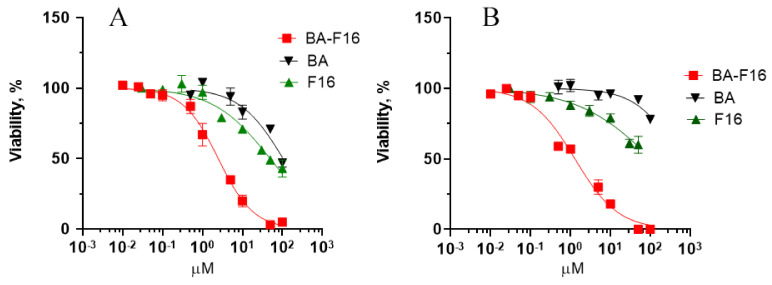
Dose dependence of the effect of compounds BA, F16, and BA–F16 on the survival of MCF-7 (**A**) and HF (**B**) cells. Cytotoxicity was assessed 48 h after the addition of substances by crystal violet indicator. Data (mean ± SEM) of 4 independent experiments are shown.

Figure 2 presents the concentration dependence of the effect of BA and its conjugate with the F16 cation on the survival of human skin fibroblasts and MCF-7 breast cancer cells. One can observed that BA and F16 cause a half-maximal inhibition of the viability of MCF-7 breast cancer cells at concentrations of 105 and 50 μM, respectively, while BA–F16 exhibits a cytotoxic effect against this cell line at a concentration of 2.1 μM (IC_50_) (Figure 2A). This suggests an increase in the cytotoxicity of the conjugate compared to the original BA by 50 times. In the case of human skin fibroblasts, we were unable to determine the IC_50_ value for BA and F16 over the concentration range used. At the same time, the BA–F16 compound, as in the case of the MCF-7 cell line, significantly suppressed the viability of human skin fibroblasts (IC_50_ = 1.5 μM) (Figure 2B). All this suggests that the penetrating F16 cation significantly enhances the cytotoxic effect of BA. However, the presented conjugate did not show increased selectivity for tumor cells compared to healthy ones. For a comparison, doxorubicin, used for the treatment of breast cancer tumors, had a cytotoxic effect on both cell lines, while the IC_50_ value was 0.6 μM for MCF-7 and 0.5 μM for HF (Figure S2).

### 3.2. BA–F16 Causes Changes in the Cell Cycle of MCF-7 and HF Cells

**Figure 3:**
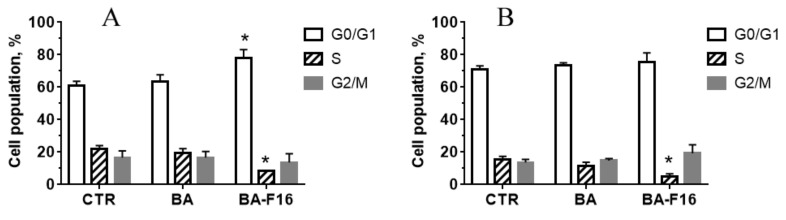
MCF-7 (**A**) and HF (**B**) cell cycle analysis after 24 h incubation with BA (5 µM) and BA–F16 (1 µM). Data are mean ± SD of three independent experiments. (* indicates *p* < 0.05 when compared with control).

In the subsequent part of the work, we evaluated the effect of BA and its conjugate with a penetrating cation on the distribution of the MCF-7 and HF cell populations over the cell cycle (Figure 3). MCF-7 and HF cells were incubated with 5 μM BA and 1 μM BA–F16 for 24 h, and analyzed by flow cytometry. At these concentrations, BA and BA–F16 did not cause cell death after 24 h of incubation, while the 3–10 μM conjugate caused a 90% death rate of the population of both cell types. BA up to 100 μM did not cause the appearance of dead cells within 24 h.

It can be observed that 5 μM BA has a significant effect on the cell cycle of both MCF-7 and human fibroblasts (Figure 3). However, the incubation of HF and MCF-7 cells with 1 μM BA–F16 resulted in an increase in the number of cells in the G_0_/G_1_ phase compared to the control cells. This increase was accompanied by a significant decrease in cell number in the S phase of the cell cycle (Figure 3). In cultured human fibroblasts, BA–F16 also resulted in an increase in the number of cells in the subG_1_ phase (Figures S3 and S4). These results suggest that BA–F16 is able to inhibit MCF-7 and HF cell viability through the inhibition of the proliferation and cell cycle arrest in the G_0_/G_1_ phase.

### 3.3. Mitochondrial Localization of BA–F16 in HF and MCF-7 Cells

**Figure 4:**
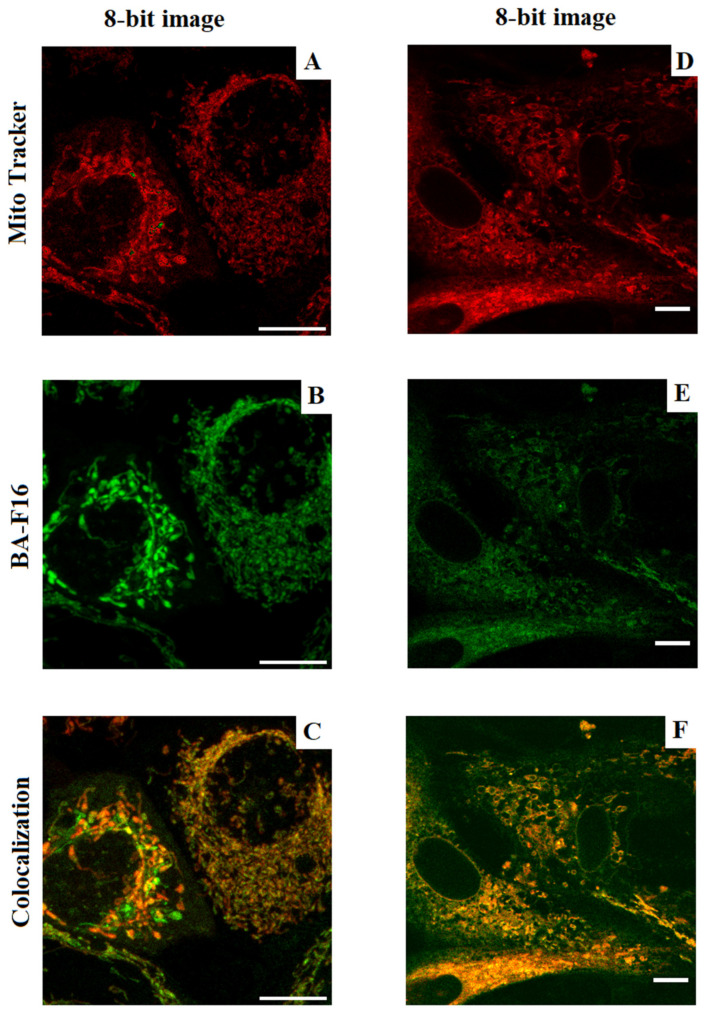
Evaluation of the mitochondrial localization of compound BA–F16 in MCF-7 breast adenocarcinoma cells (**A**–**C**) and human fibroblasts (**D**–**F**) by confocal microscopy. (**A**,**D**) MitoTracker Red fluorescence intensity image (200 nM, (λex/λem) = 638/650 nm), (**B**,**E**) F16 fluorescence intensity image (200 nM, (λex/λem) = 450/550 nm), (**C**,**F**) image obtained by overlaying images “A” and “B”, “D” and “E”. Scale bars: 10 µm.

In our previous work, we demonstrated that conjugates of BA with the penetrating F16 cation are able to actively interact with isolated mitochondria and influence their functioning [18]. In this study, we continued to study the interaction of this class of compounds with mitochondria and cells. In this regard, one of the objectives of the present study was to evaluate how these compounds (BA–F16) are able to accumulate in the mitochondria of healthy cells (human skin fibroblasts) and MCF-7 breast cancer cells. Since the penetrating F16 cation fluoresces, the degree of accumulation of the conjugate in mitochondria could be determined by the colocalization of the F16 cation with the MitoTracker DeepRed probe. Figure 4 presents representative images of human skin fibroblast cells and MCF-7 loaded with 200 nM MitoTracker DeepRed and conjugate. Processing of the obtained results made it possible to determine that 16.1 ± 3.1% of human fibroblast mitochondria stained with MitoTracker DeepRed and 24.2 ± 1.8% (*p* < 0.05) of MCF-7 cell mitochondria contained the tested conjugate.

### 3.4. BA–F16 Stimulates the Production of Reactive Oxygen Species in MCF-7 and HF Cells

**Figure 5:**
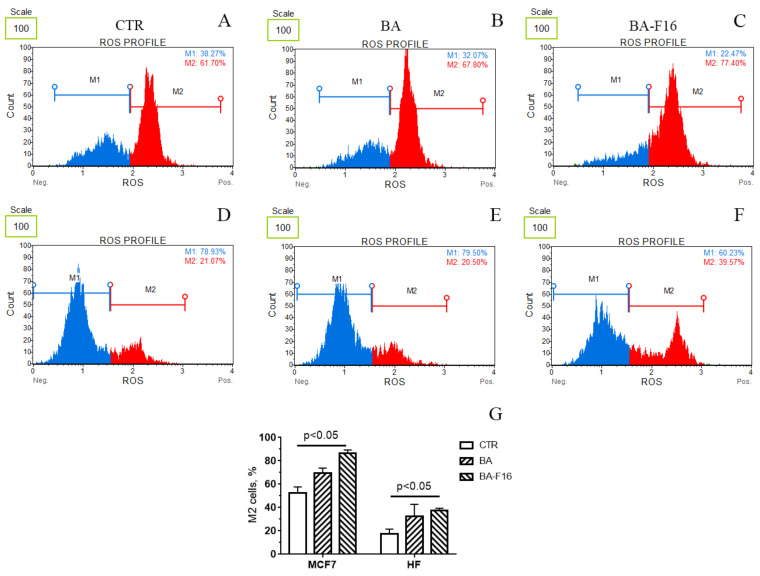
Effect of 5 μM of BA and 1 μM of BA–F16 on the production of reactive oxygen species by MCF-7 and HF cells. (**A**–**F**) Typical distribution diagrams of MCF-7 (**A**–**C**) and HF (**D**–**F**) cell populations in the presence of BA (**B**,**E**) and BA–F16 (**C**,**F**). (**G**) Calculation of ROS-positive cells (%) in experimental groups. Means ± SD are shown (*n* = 4–5).

The cytotoxic effect of mitochondria-targeted compounds is believed to be associated with a prooxidant effect [13,18]. In this study, we evaluated the effect of BA and its conjugate with a penetrating cation on HF and MCF-7. Figure 5 presents typical flow cytometry data for the number of ROS-positive and ROS-negative cells in a culture of MCF-7 cells (A–C), and human fibroblasts (D–F) in the absence and presence of 5 μM BA and BA–F16. One can observed that the number of ROS-positive cells (the proportion of cells in which superoxide-induced fluorescence belongs to the M2 peak) in the MCF-7 culture is significantly higher than in the human fibroblast culture, which corresponds to the literature data. The incubation of both cell lines with BA (Figure 5B,E,G) did not result in a significant increase in superoxide anion generation. At the same time, the pre-incubation of the cells with 1 μM of BA–F16 shifts the profile of ROS formation towards ROS-positive cells, which indicates a considerable of superoxide in the cells in the presence of this conjugate (Figure 5C,F,G).

### 3.5. BA–F16 Increases the Permeability and Fluidity of Liposomal Membranes

**Figure 6:**
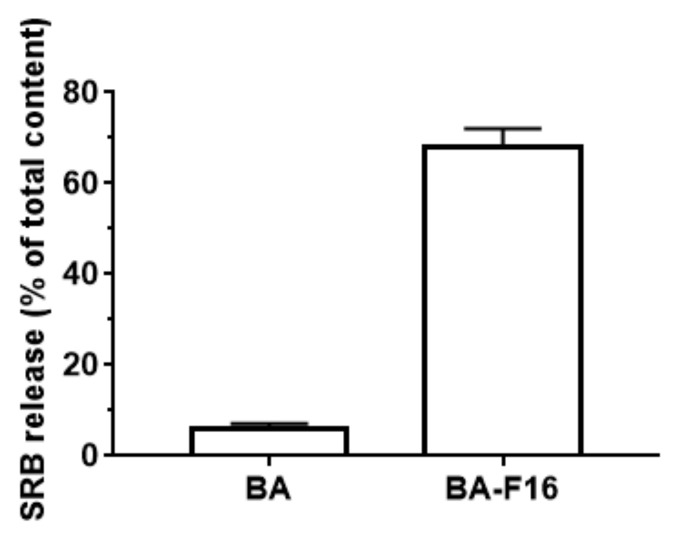
BA–F16 (1 μM) but not BA (50 μM) induces the release of the sulforodamine B dye from unilamellar lecithin liposomes. The incubation medium contained: 10 mM Tris-HCl (pH 7.5), 40 mM KCl, and 50 μM EGTA. The results are presented as means ± SEM (*n* = 5).

One of the factors affecting cytotoxicity is an increase in the nonspecific permeability of cell membranes. In this regard, we investigated the effects of BA and BA–F16 on the permeability of lecithin liposomes loaded with sulforhodamine B dye. One can observed that 1 μM of BA–F16 induces a significant release of sulforodamine B from the liposomes. Unlike BA–F16, 50 μM of BA is ineffective as a permeabilizing agent (Figure 6).

**Figure 7:**
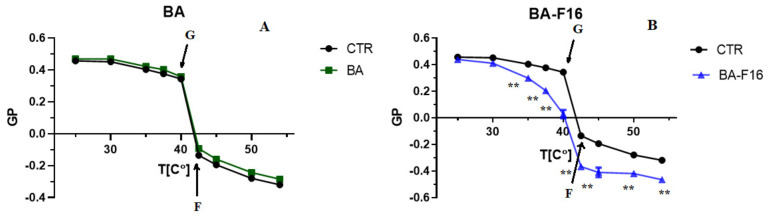
Effect of 10 µM of BA (**A**) and 2 µM of BA–F16 (**B**) on the phase state of DPPC liposomes. The transition from a solid- (G—gel state) to a liquid-crystalline state (F—fluid state) is defined as the point of maximum curvature. The incubation medium contained: 10 mM Tris-HCl (pH 7.5), 40 mM KCl, and 50 μM EGTA. Temperature measurements range from 25 to 54 °C. CTR—without additions. The results are presented as means ± SEM (*n* = 3). ** indicates *p* < 0.01 when compared with control curve.

To identify the cause of the increase in liposome membrane permeability, we evaluated the effects of BA and BA–F16 on the state of DPPC membranes using the fluorescent probe laurdan (Figure 7).

Laurdan is an environment-sensitive fluorescent probe, which is conventionally used for monitoring the phase state of lipid membranes. The generalized polarization (GP) of laurdan—an indicator defined as the relative difference between fluorescence intensities at two wavelengths (red and blue peaks)—reflects the hydration of the lipid bilayer and the mobility of water molecules in the region of lipid heads [23]. One can be observe that up to 42 °C (the DPPC phase-transition point), the GP parameter is in the zone of positive values. This indicates that the membrane is in an ordered state (gel phase). Points G and F present the temperature range of the phase transition of lipids from gel (gel state) to liquid-crystalline (fluid state) phases. A further increase in the temperature leads to a gradual decrease in the parameter GP, which is in the zone of negative values (liquid-crystal phase). A total of 10 μM of BA has no effect on the phase state of the membrane of liposomes formed from DPPC over the entire temperature range studied (Figure 7A). At the same time, when 2 μM of BA–F16 is added to DPPC liposomes, the phase transition start point shifts to the left and the phase transition temperature of the lipids increases (smoothing of the graph curvature) (Figure 7B). At a temperature higher than the phase transition point, a decrease in the GP parameter can be observed. All this suggests that BA–F16 has a pronounced membranotropic effect, resulting in the liquefaction of the liposomal membranes.

## 4. Discussion

Pentacyclic triterpenoids, such as BA, are known to be effective antitumor compounds with relatively selective cytotoxic activity against various cancer cell lines and tumors [24,25,26]. BA has a cytotoxic effect in vitro at micromolar concentrations [25]; however, it has a limited solubility in water, which requires its preliminary derivatization to improve its pharmacological characteristics. An effective strategy for increasing the therapeutic effect of triterpenes is their conjugation with molecules that have target properties and, in particular, a mitochondrial orientation. Indeed, recently synthesized hybrids of triterpenes and a mitochondria-targeted delocalized F16 cation have significantly higher antitumor activity and showed an approximately 200-fold decrease in IC_50_ values compared to BA, and an 800-fold decrease in IC_50_ values compared to F16 [17].

An important task in the development of antitumor agents is to elucidate the selectivity of their action with respect to targets localized in cancer cells. Previously, we showed that newly synthesized derivatives of BA and F16 have a toxic effect on healthy cells and, in particular, thymocytes, which is due to a significant inhibition of the functional activity of mitochondrial OXPHOS and ROS burst, as well as the permeabilization of the lipid bilayer of their membranes [18]. Additional comparative studies are needed to clarify the prospects for the use of BA and F16 conjugates as antitumor agents. Therefore, in this study, we evaluated the selectivity of the cytotoxic effects of one of the most effective conjugates on cancerous (MCF-7 breast cells) and healthy (human skin fibroblasts) cells, its effect on the cell cycle of these cell lines, studied their ability to accumulate in mitochondria, and also determined the effect of the conjugate on the ROS status of the cells. In addition, using liposomes, we evaluated the effect of the conjugate on the phase state of the lipid bilayer of membranes and their permeabilization.

At the first stage of the study, we confirmed that the presence of a delocalized F16 cation in the hybrid structure containing BA significantly increased its cytotoxic properties (Figure 2). However, if for the original BA it can be concluded that there is selectivity to MCF-7, then in the case of the conjugate, we did not observe significant differences in IC_50_ between cancer and healthy cells. The toxicity of the conjugate was comparable to that of doxorubicin, a widely used antitumor drug, whose IC_50_ was near 0.5 μM for both studied cell lines. Interestingly, the F16 cation presented a significant toxic effect against MCF-7 cells (IC_50_ = 50 μM). This effect was even stronger than that of BA. However, we were unable to determine the IC_50_ of F16 for healthy fibroblasts. Although, in the case of these cells, a toxic effect was observed.

Triterpene-F16 conjugates have previously been shown to induce cell cycle arrest in cancer cells. In this study, we observed that the tested BA–F16 conjugate also caused cell cycle arrest in both cancerous and healthy cells in the G_0_/G_1_ resting phase (Figure 3). Previously, it was shown that blocking the MCF-7 cell cycle in the G_0_ phase leads to a significant decrease in cell proliferation [27,28]. One could assume that a similar mechanism occurred in the present case. It should be noted that the studied conjugate also caused an increase in the proportion of fibroblasts in the subG_1_ phase, which may indicate a change in chromatin levels and the preparation of cells for death [29].

The presence of fluorescent properties made it possible to quantify the mitochondrial localization of the conjugate in the studied cell lines (Figure 4). We observed that approximately the same part of the mitochondria in the cells (about 16–23%) absorbed the conjugate. At the same time, the conjugate demonstrated slightly more pronounced selectivity for cancer cells.

Previously, we showed that some conjugates of betulinic acid with F16 induced a high generation of superoxide radical in rat thymocytes, whose source was mitochondria. The pro-oxidant effect of the agent was also confirmed on isolated mitochondria and, apparently, was due to the inhibition of the activity of complex I of the respiratory chain of organelles [18]. In this study, we also compared the ROS status of healthy and cancer cells under the same incubation conditions, and the effect of the conjugate on the change in this parameter (Figure 5). The culture of MCF-7 cells is characterized by a large proportion of ROS-positive cells compared to healthy fibroblasts, which corresponds to the literature data and is due to the important role of ROS in cancer cell proliferation and the activation of mitogenic transcription factors [30,31]. In this case, the conjugate, in contrast to the parental BA, caused a significant increase in the level of ROS-positive cells in both cell lines, which confirms the pro-oxidant nature of the studied agent. At the same time, the relative ROS burst effect of the agent was approximately the same in the case of healthy and cancerous cells.

In our previous work, it was shown that the use of a triethylene glycol spacer to bind F16 and BA led to a pronounced ability of the conjugate to permeabilize lipid membranes, including mitochondrial ones [18]. The use of triethylene glycol as a linker has also been shown to increase the membranotropic properties of triphenylphosphonium cations of the diterpenoid isosteviol [32]. In the case of the F16 conjugate and BA, this effect was associated with significant rearrangements of liposome membranes, as indicated by their separation into populations of different sizes and the appearance of giant vesicles, which is also accompanied by the release of almost 70% of the fluorescent SRB probe loaded into the vesicles (Figure 6). At the same time, native BA is practically ineffective. It could be assumed that the membranotropic effect of the studied conjugate could be caused by a change in the order of lipids in the liposome membrane. To test this hypothesis, we assessed the effects of the conjugate and native BA on lipid ordering in unilamellar liposome membranes using a laurdan fluorescent probe. Additionally, if BA did not affect the fluorescence spectrum of laurdan and, accordingly, the phase state of liposome membranes (Figure 7A), then BA–F16 caused an increase in the temperature of the lipid phase transition (smoothing the curvature of the graph) (Figure 7B), which indicates the liquefaction of liposome membranes. This confirms that the conjugate of BA and F16 containing a triethylene glycol spacer has pronounced membranotropic properties and is able to permeabilize lipid membranes, including cell and organelle membranes, contributing to the induction of various types of cell death.

As shown in our previous work, the BA–F16 compound exhibited a 10-fold selectivity with respect to some cancer cell lines [17]. Unfortunately, in this study, we did not obtain the selectivity of this conjugate against the MCF-7 line of breast cancer cells. Additional studies are needed to make the toxic selectivity of betulinic acid conjugates with delivery molecules higher with respect to cancer cells. Based on the results obtained, it can be observed that the F16 cation itself is toxic to both cancerous and healthy cells. It is possible that the use of penetrating cations showing less toxicity to healthy cells will help increase the selectivity of betulinic acid and its conjugate against cancer cells.

## 5. Conclusions

The data obtained suggest that the introduction of a mitochondria-targeted cation through a triethylene glycol spacer significantly increases the cytotoxic and membranotropic effects of BA both in relation to breast cancer cells and healthy fibroblasts, which is also accompanied by blocking cell proliferation. The effect, apparently, is due to the selective accumulation of the conjugate in mitochondria, which is more pronounced in cancer cells. It can be assumed that the cytotoxicity of the conjugate is associated with the hyperproduction of ROS, which is observed both in cancerous and healthy cells. The mechanism of action of the conjugate may be due to its ability to permeabilize cell membranes and their organelles, which contributes to cytotoxicity. The studied conjugate did not have significant selective cytotoxicity, at least against the studied line of MCF-7 breast cancer cells in comparison with normal human fibroblasts (HFs), and additional modifications of the structure of the conjugates are necessary to provide them with selective properties and reduce the cytotoxicity for healthy cells.

## Data Availability

The data presented in this study are available on request from the corresponding author.

## Supplementary Materials

The following supporting information can be downloaded at: https://www.mdpi.com/article/10.3390/biomedicines10112903/s1, Figure S1: Synthesis of conjugate BA-F16.; Figure S2: Viability of MCF-7 and HF cells after 48 h treatment with doxorubicin.; Figure S3: MCF-7 cell cycle analysis after 24-h incubation with BA (5 µM) and BA-F16 (1 µM); Figure S4: HF cell cycle analysis after 24-h incubation with BA (5 µM) and BA-F16 (1 µM).
