# Supplementary material for: Comparative Study of Cytotoxic and Membranotropic Properties of Betulinic Acid-F16 Conjugate on Breast Adenocarcinoma Cells (MCF-7) and Primary Human Fibroblasts
Source: Biomedicines. 2022 Nov 11;10(11):2903. doi: 10.3390/biomedicines10112903 (PMC9687851; doi:10.3390/biomedicines10112903)
Supplement: Supplementary file 1 [file biomedicines-10-02903-s001.zip › biomedicines-1973248-supplementary.pdf]

Supplementary Materials

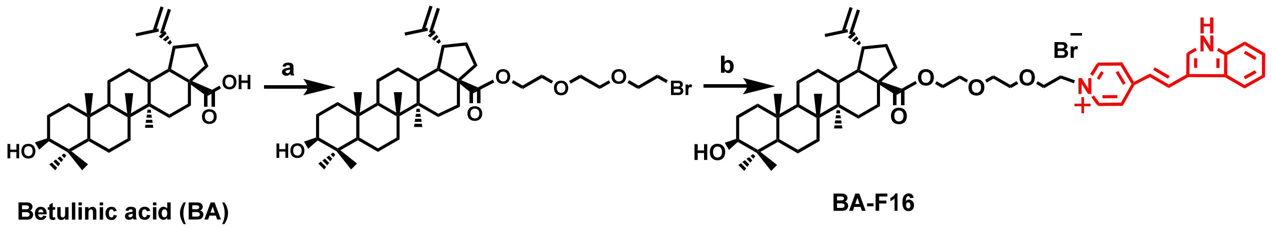

Betulinic acid (BA)

Figure S1. Synthesis of conjugate **BA-F16**. Reagents and conditions: **a** tri(ethylene glycol) dibromide, K_2CO_3, DMF, 50 °C, 2h; **b** (E)-4-(2-(1*H*-indol-3-yl)vinyl)pyridine, DMF, 85 °C, Ar, 12 h.

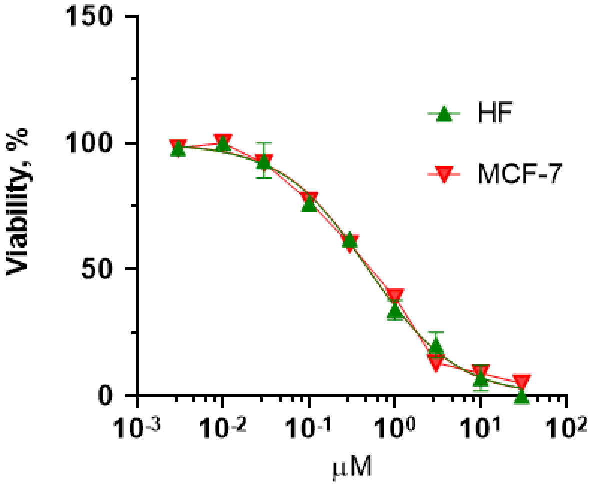

Figure S2. Viability of MCF-7 and HF cells after 48 hours treatment with doxorubicin. Cytotoxicity was assessed 48 h after the addition of substances by crystal violet indicator. Data (mean ± SEM) of 4 independent experiments are shown.

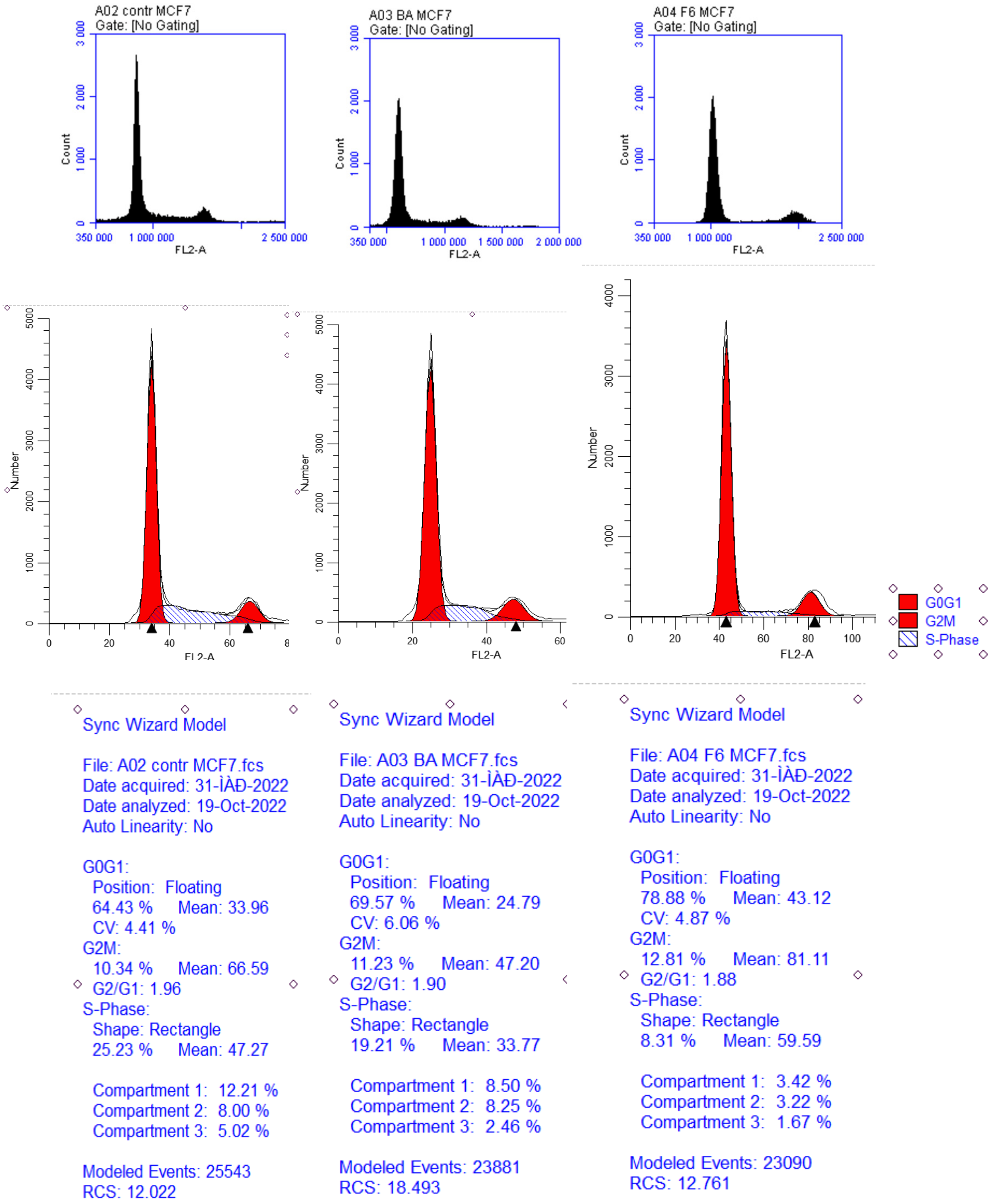

Figure S3. MCF-7 cell cycle analysis after 24-h incubation with BA (5 µM) and BA-F16 (1 µM). Results shown are one of three independent experiments conducted. Upper panel – representative figures of cell cycle distribution (G0/G1, S, and G2/M). Bottom panel – results of cell cycle distribution evaluated using ModFit LT 4.1 software. In the figure, the designation F6 corresponds to the BA-F16 conjugate.

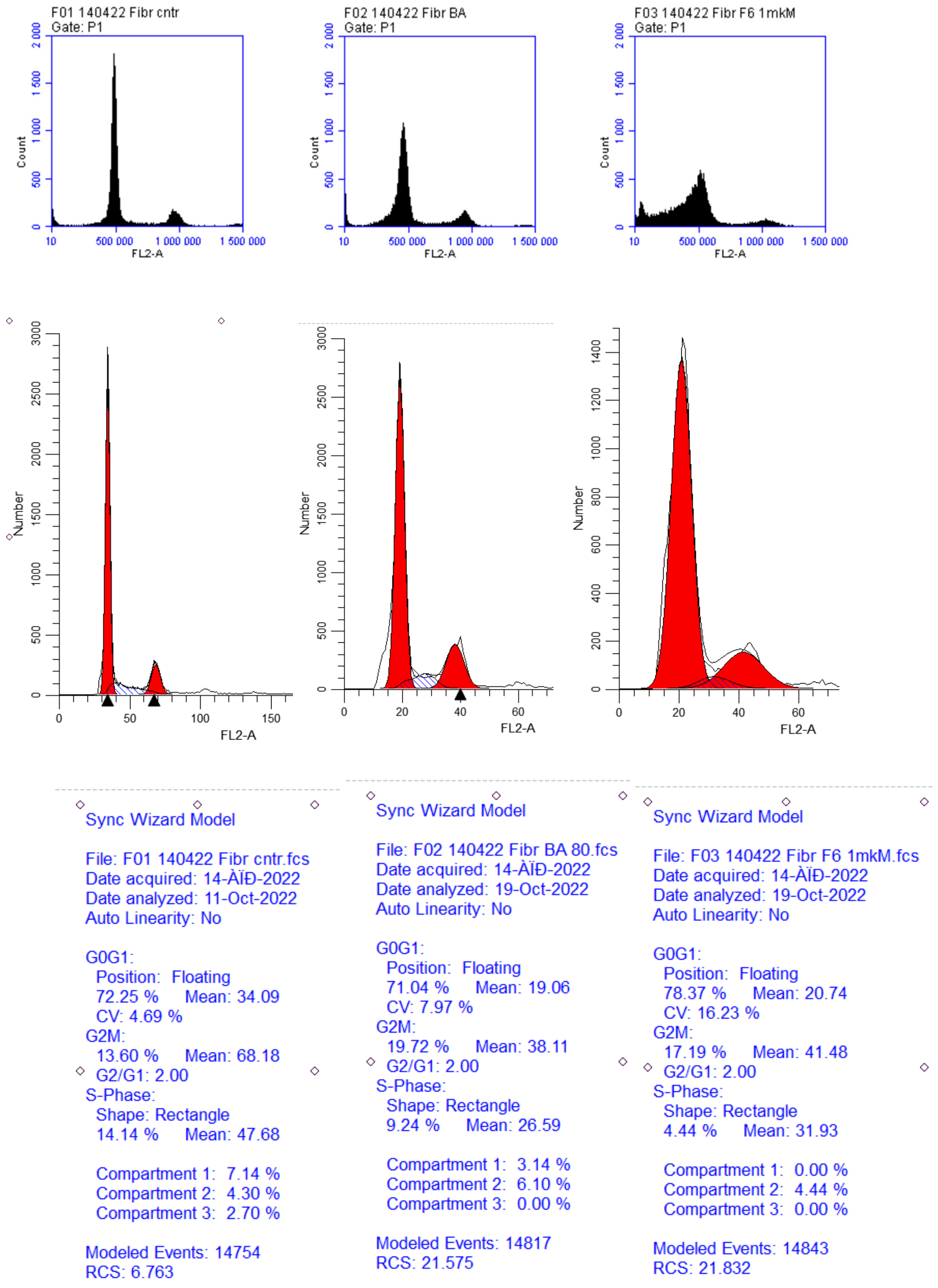

Figure S4. HF cell cycle analysis after 24-h incubation with BA (5 μM) and BA-F16 (1 μM). Results shown are one of three independent experiments conducted. Upper panel – representative figures of cell cycle distribution (G0/G1, S, and G2/M). Bottom panel – results of cell cycle distribution evaluated using ModFit LT 4.1 software. In the figure, the designation F6 corresponds to the BA-F16 conjugate.
